# Advancing Plant Microbiome Research Through Host DNA Depletion Techniques

**DOI:** 10.1111/pbi.70379

**Published:** 2025-10-13

**Authors:** Yao Wang, Junbo Yang, Huiyu Hou, Luyang Song, Xu Cheng, Yong‐Xin Liu

**Affiliations:** ^1^ Genome Analysis Laboratory of the Ministry of Agriculture and Rural Affairs, Agricultural Genomics Institute at Shenzhen, Chinese Academy of Agricultural Sciences Shenzhen China; ^2^ Yazhouwan National Laboratory Sanya China; ^3^ College of Plant Protection Henan Agricultural University Zhengzhou China

**Keywords:** host DNA depletion, metagenomics, microbial enrichment, microbiome‐based agriculture, plant microbiome

## Abstract

Plants provide ecological habitats for diverse microorganisms, making accurate metagenomic sequencing essential for understanding the complex interactions that support plant growth, development and disease resistance. However, host DNA contamination poses a major challenge in plant microbiome studies, obscuring microbial genetic signatures and complicating the accurate analysis of microbial genomes. This review provides a comprehensive overview of current host DNA depletion strategies, including physical separation (e.g., filtration, gradient centrifugation), selective lysis and enzymatic treatments targeting plant cell walls. Advanced techniques such as targeted sequence capture with magnetic beads, methylation‐based enrichment and nanopore selective sequencing offer additional options for host DNA removal. Despite these advances, current methods still face challenges in efficiency, specificity and applicability, emphasising the need for tailored strategies and the exploration of novel approaches for microbial enrichment. Innovations like CRISPR‐Cas9 and chromatin immunoprecipitation‐based host DNA depletion methods are proposed to provide novel directions for addressing current limitations. The development and refinement of host depletion techniques tailored to plant systems are crucial for enabling high‐resolution, cost‐effective metagenomic studies. These efforts promise to deepen our understanding of microbial diversity and functionality, ultimately accelerating microbiome‐based innovations in crop improvement, sustainable agriculture and ecosystem resilience.

## Graphical abstract

The plant microbiome plays an essential role in promoting plant growth, enhancing stress tolerance and maintaining overall plant health. Metagenomic technologies have significantly expanded our ability to investigate plant–microbe interactions at high taxonomic and functional resolutions. However, the application of metagenomics in plant microbiome studies is challenged by the high ratio of host to microbial DNA in plant‐derived samples. Excessive host DNA often overwhelms microbial signals, complicating microbial genome recovery and functional analysis. This review highlights host DNA depletion strategies to enhance microbial detection and functional analysis in metagenomic sequencing. These approaches are categorised into three major groups: (1) Cell Size‐Based Separation: Techniques such as gradient centrifugation, differential lysis and enzymatic hydrolysis exploit structural and size differences between plant and microbial cells to physically separate microbial content from plant material. (2) Sequence Difference‐Based Methods: Targeted sequence capture, digestion using restriction enzymes and selective nanopore sequencing distinguish microbial DNA from host DNA based on sequence‐specific features, selectively enriching microbial reads. (3) Proposed Novel Molecular Strategies: Potential methods including CRISPR‐Cas9‐mediated host genome cleavage and chromatin immunoprecipitation leverage molecular and chromatin structure differences to selectively remove host DNA, thereby preserving and enriching microbial DNA. Collectively, these approaches aim to reduce host DNA contamination, thereby improving the sensitivity, accuracy and cost‐effectiveness of plant metagenomic analyses. Implementing efficient host depletion strategies is crucial for obtaining deeper insights into plant–microbe interactions and accelerating the discovery of beneficial microbes for sustainable agricultural applications.

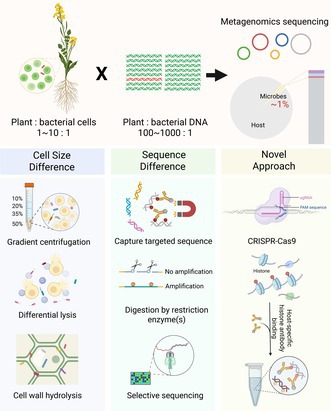

## 1 Introduction

Plants are colonised by a wide variety of microorganisms—including bacteria, archaea, fungi, viruses and protists—that inhabit diverse niches such as the rhizosphere, endosphere and phyllosphere (Hardoim et al. 2015; Russ et al. 2023; Trivedi et al. 2020; Vorholt 2012). These plant‐associated microbiomes form intricate networks of interactions with their hosts, contributing to nutrient acquisition, growth promotion, stress tolerance and defence against pathogens (Durán et al. 2018; Finkel et al. 2020; Kwak et al. 2018; Salas‐González et al. 2021; Zhang et al. 2025). In turn, plants modulate microbial communities through root exudates, immune signalling and habitat structuring, leading to dynamic, reciprocal relationships that are essential for plant health and ecosystem functioning (Huang et al. 2019; Lebeis et al. 2015; Liu et al. 2021; Lv et al. 2024; Song et al. 2021; Wang and Song 2022). The notion of plant ‘holobiont’ was introduced to emphasise that plant health and evolution are not determined by the plant alone, but rather by the collective interaction between the host and its associated microbial communities (Vandenkoornhuyse et al. 2015; Zilber‐Rosenberg and Rosenberg 2008). Far from being passive inhabitants, microbial partners are essential contributors to plant function and environmental adaptation, forming a co‐dependent system that responds holistically to biotic and abiotic stimuli (Hassani et al. 2018).

The interactions between plants and microorganisms encompass a wide range of ecological relationships, including mutualism, commensalism and pathogenicity (Hassani et al. 2018; Shi et al. 2024). Beneficial microbes, such as plant growth‐promoting rhizobacteria (PGPR) and mycorrhizal fungi, contribute to plant health by enhancing nutrient uptake, producing phytohormones and suppressing pathogens (Jiang et al. 2017; Liu et al. 2025). Commensal microorganisms typically do not exert direct effects on the host but can influence plant physiology indirectly by modulating immune responses or interacting with other microbial taxa (Berendsen et al. 2012; Newton et al. 2010; Santoyo 2022). In contrast, pathogenic microbes can disrupt plant health through tissue invasion, competition for resources and the release of phytotoxins (Wang et al. 2023; Xu et al. 2022). Moreover, some microbial taxa may also facilitate pathogen germination, colonisation, or act as latent pathogens, constituting a ‘pathobiome’ that disrupts host health (Bass et al. 2019; Lv et al. 2023; Wang et al. 2023). Together, these multifaceted interactions constitute the plant microbiome, a dynamic consortium that is increasingly recognised as a key determinant of plant productivity and resilience (Fitzpatrick et al. 2020).

A deeper understanding of the plant microbiome holds tremendous potential for advancing sustainable agriculture, offering nature‐based solutions for crop improvement, biocontrol, soil fertility and climate resilience (Gouda et al. 2018; Mendes et al. 2011; Nerva et al. 2022). However, fully leveraging this potential requires moving beyond simple taxonomic inventories to uncover the functional traits and ecological dynamics of microbial communities (Levy et al. 2018). The advent of metagenomics has revolutionised our ability to study the plant microbiome beyond traditional culturing approaches. By directly extracting and sequencing DNA from environmental samples, plant metagenomics provides a comprehensive, high‐resolution view of microbial community structure, taxonomic composition and functional potential (Quince et al. 2017; Saheb Kashaf et al. 2021). It has enabled the reconstruction of metagenome‐assembled genomes (MAGs), discovery of novel microbial lineages, and identification of microbial genes involved in key ecological functions such as nitrogen fixation, siderophore biosynthesis, phytohormone metabolism and antimicrobial compound production (Howe et al. 2023; Xu et al. 2021; Zhang et al. 2019). For instance, Bulgarelli et al. (2015) demonstrated that both wild and domesticated barley shape distinct root‐associated microbiota with enriched functions related to pathogenesis, secretion and nutrient mobilisation. Shi, Li, et al. (2019) revealed that the composition and functional traits of the geocaulosphere soil microbiome are closely associated with potato common scab severity, highlighting potential interactions between antagonistic and pathogenic microorganisms. More recently, Chang et al. (2025) highlighted that rice domestication reduced nitrogen‐fixing microbial functions and promotes N_2_O‐producing microbes in the rhizosphere.

Despite these advances, the application of metagenomics to plant microbiome research is still limited by several technical obstacles. A major technical obstacle is the overwhelming abundance of host‐derived DNA in plant samples, particularly in low‐biomass compartments such as the phyllosphere or endosphere (Yeoh 2021). As a result, microbial reads are often masked by host sequences, leading to inefficient use of sequencing resources, reduced detection sensitivity and potential bias in microbial community reconstruction (McArdle and Kaforou 2020; Nearing et al. 2021). While increasing sequencing depth can partially overcome this problem, it is cost‐prohibitive and computationally demanding, especially for large‐scale studies (Pereira‐Marques et al. 2019).

To overcome this limitation, various host DNA depletion techniques have been proposed, including physical separation methods (e.g., filtration, gradient centrifugation), selective lysis and enzymatic degradation of host cells and sequence‐based strategies such as CRISPR‐Cas9 cleavage and adaptive nanopore sequencing. While many of these approaches have been optimised in mammalian microbiome studies (Islam Sajib et al. 2024; Shi et al. 2022), their application to plant systems remains technically challenging due to differences in tissue structure, genome size and cell wall composition.

In this review, we provide a comprehensive overview of host DNA removal strategies in the context of plant metagenomics, evaluate their advantages and limitations and highlight innovative molecular tools for microbial enrichment. By drawing lessons from both plant and mammalian studies, we aim to guide future developments in this rapidly evolving field. Improving host depletion will enable more accurate and cost‐effective profiling of plant‐associated microbiota, thereby advancing our understanding of plant–microbe interactions and supporting the development of microbiome‐based solutions for sustainable agriculture.

## 2 The Importance of Host Depletion Methods in Plant Metagenomics

**FIGURE 1:**
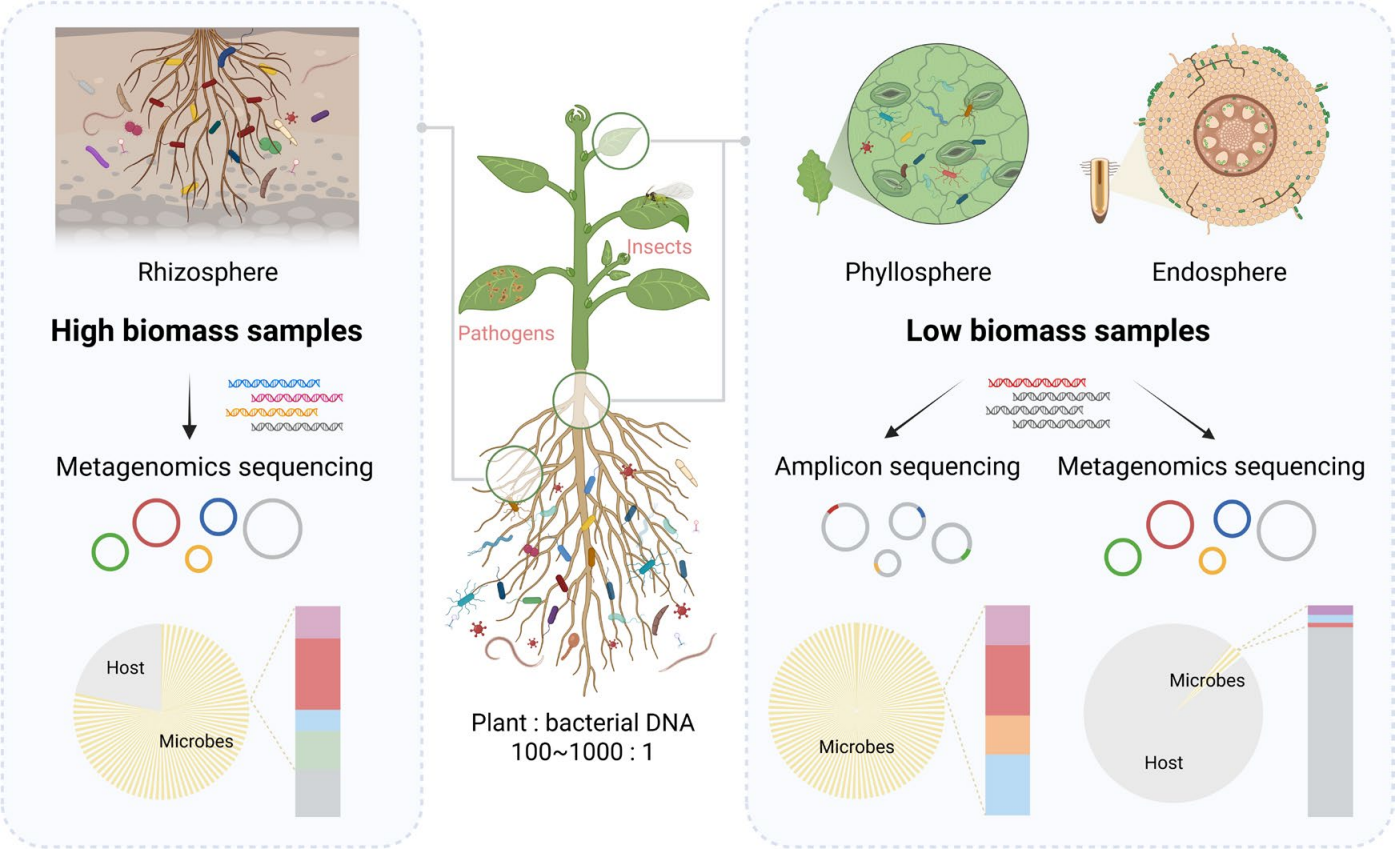
Importance of host depletion methods in plant metagenomics. Understanding the complex interactions between plants and their associated microbial communities is pivotal for plant health and growth. Metagenomic analysis is a powerful tool to reveal the composition, structure and functional potential of microbial communities. As research focus has gradually expanded from rhizosphere soil to specific plant compartments such as roots, stems and leaves, and from high biomass to low biomass samples, the severe host contamination has become a significant issue. A high proportion of host DNA results in wasted sequencing resources, reduced sensitivity and obscured microbial signals, thereby impeding the effective application of metagenomics in plant microbiome research. The development and optimisation of host depletion approaches are therefore essential to improve microbial signal recovery, enhance sequencing efficiency and facilitate deeper insights into plant–microbe interactions.

Research into plant microbiomes is pivotal for understanding the intricate symbiosis between plants and their microbial communities, driving advancements in agricultural innovation, ecosystem sustainability and environmental conservation (Li et al. 2024; Trivedi et al. 2021). Microbes inhabiting the phyllosphere, rhizosphere and endosphere play vital roles in nutrient cycling, disease resistance and stress tolerance, underscoring their importance in plant health and development (Castrillo et al. 2017; Compant et al. 2024; Wang et al. 2024). Plant microbiome studies have predominantly focused on descriptive analyses, heavily relying on 16S rRNA gene amplicon sequencing (Bai et al. 2015; Carlström et al. 2019; Edwards et al. 2015). While these studies have offered valuable insights into microbial diversity, their reliance on specific primers limits the resolution and falls short in providing functional perspectives. There is growing recognition of the need to shift from descriptive to more functional and mechanistic studies. Research has expanded from the rhizosphere to specific plant compartments such as roots, stems and leaves, and from high‐biomass to low‐biomass samples (Figure 1). However, this shift presents new challenges, particularly the issue of high host DNA contamination in metagenomic sequencing of plant‐associated microbiomes (Dai et al. 2025).

Plant genomes are substantially larger than microbial genomes (e.g., the oilseed rape genome is approximately 1.1 Gb, compared to an average bacterial genome size of ~3.6 Mb) (Chalhoub et al. 2014). Even a small amount of plant‐derived material can overwhelm the microbial DNA pool, leading to insufficient sequencing coverage of microbial genomes and impeding the accurate detection of microbial diversity and functional genes. Although host DNA can be filtered out post‐sequencing using bioinformatics tools (Langmead and Salzberg 2012; Lu et al. 2022; Rumbavicius et al. 2023), the presence of host‐derived sequences results in wasted sequencing resources, reduced sensitivity, obscured microbial differences and even overlooked signals from target microbes in trace amounts (Angel et al. 2025; Kok et al. 2022; Rubiola et al. 2020). Increasing sequencing depth (i.e., the number of reads generated per sample) can enhance the representation of microbial reads in the metagenome (Pereira‐Marques et al. 2019). However, the associated high sequencing costs, data analysis time and computational demands exceed the capacity of many laboratories, limiting the feasibility of large‐scale analyses. Overcoming host DNA contamination is therefore critical for enriching microbial content, optimising sequencing efficiency and obtaining high‐quality microbial genome data. Addressing these challenges will not only deepen our understanding of plant–microbe interactions but also unlock the potential for discovering functional genes and beneficial microbes, ultimately advancing sustainable agricultural practices and environmental stewardship.

## 3 Advances in Host Depletion Methods

To mitigate host DNA contamination, various host depletion methods have been developed according to cellular and sequence differences between host and microorganisms. However, most published and commercial solutions are tailored for mammalian samples (Marchukov et al. 2023; Shi et al. 2022), with a lack of effective strategies to efficiently obtain abundant microbial reads from plant tissue samples. Here, we review the host depletion strategies currently employed in plant research, while drawing parallels to the more established techniques used in mammalian studies. By doing so, we aim to inspire innovative methods tailored specifically to the unique challenges of plant microbiome research.

### 3.1 Microbial Enrichment Prior to DNA Extraction

**FIGURE 2:**
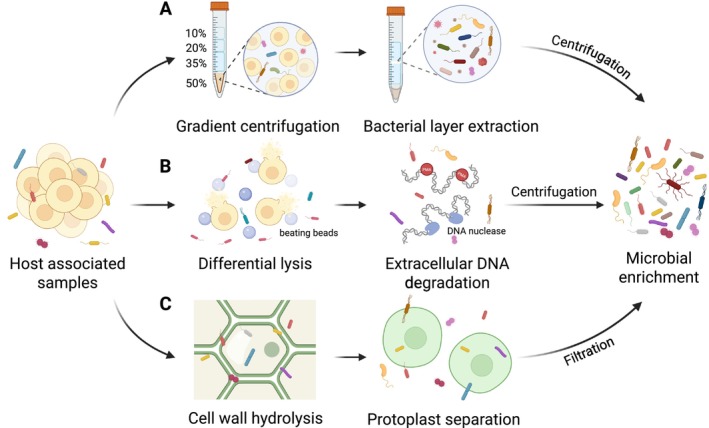
Microbial enrichment methods for host‐associated samples. Before DNA extraction, various techniques can be explored to separate host cells from microbial cells and improve microbial enrichment. (A) Density gradient centrifugation is employed to separate bacterial cells by density differences, with bacterial layers subsequently extracted from specific gradient fractions for further processing. (B) Differential lysis by chemicals or mechanical beating targets host cells to release host DNA while minimising bacterial cell damage, followed by extracellular DNA degradation or propidium monoazide (PMA) treatment to diminish host DNA contamination. (C) Plant cell walls are selectively hydrolysed by enzymes, such as cellulase, hemicellulase and pectinase without harming microbial cells to facilitate downstream microbial DNA extraction.

By exploiting the differences in the size and density between microorganisms and eukaryotic host cells, techniques such as filtration, differential centrifugation and density gradient centrifugation have been commonly used to separate host cells from microbial cells (Figure 2A) (Hevia et al. 2015; Ikeda et al. 2009; Masuda et al. 2024; Utturkar et al. 2016). For instance, density gradient centrifugation successfully enriched endophytic microbes from sugar beet, achieving approximately 24.6% non‐host DNA content after quality filtering and host DNA removal via mapping (Carrión et al. 2019). However, while gradient centrifugation can effectively reduce host contamination and enrich the microorganisms, it also diminishes the overall microbial content and can result in biased enrichment of certain microbial populations. Moreover, it requires a large sample input, rendering it unsuitable for rare or low‐biomass samples (Chapelle et al. 2015). Furthermore, rinsing with buffer or combined with sonication is also commonly employed for enriching the phyllosphere microbes. Rice phyllosphere was collected through sonication in a buffer to obtain metagenome‐assembled genomes (MAGs) (Su et al. 2022). However, excessive sonication may disrupt both host and microbial cells, leading to host contaminations and potential bias for the recovery of the microbial community; therefore, the frequency and energy input need to be fine‐tuned. These approaches can be adapted for specific samples as a pre‐treatment to separate host and microbial cells.

Beyond these conventional approaches, several advanced strategies may provide new opportunities for microbial enrichment in plant‐associated samples. Acoustophoresis, primarily applied in blood‐based samples, utilises ultrasonic standing waves in microfluidic channels to separate particles based on their size, density and compressibility (Ohlsson et al. 2018; Wu et al. 2019). Microfluidics‐based approaches further exploit microscale fluid dynamics and forces such as acoustics or dielectrophoresis to manipulate and sort microbial cells with high precision (Lazarevic et al. 2022; Yu et al. 2022). Single‐cell technologies, including fluorescence‐activated cell sorting (FACS) and droplet‐based microfluidics, enable the isolation of individual microbial cells for downstream genome amplification (McCully et al. 2023; Utturkar et al. 2016). These approaches have been successfully applied to recover uncultured and low‐abundance taxa from complex communities, providing opportunities to resolve functional heterogeneity and host–microbe interactions at single‐cell resolution (Aleklett et al. 2018; Pryszlak et al. 2022; Shi, Shao, et al. 2019). Moreover, immunomagnetic separation uses antibodies or synthetic ligands immobilised on paramagnetic beads to selectively capture microbial taxa, a strategy widely applied in food safety that could be adapted to selectively enrich known taxa in plant microbiome samples (Wang et al. 2020). Importantly, most of these methods require samples to be processed into cell suspensions through homogenisation or enzymatic digestion. Such preprocessing introduces additional challenges, including the simultaneous release of host material and potential loss of fragile microbes, underscoring the need for protocol optimisation in plant systems. Additionally, laser microdissection (LMD) provides a spatially resolved strategy by precisely excising microbe‐rich host tissue domains under microscopic visualisation. Sites of interest can be isolated to reduce bulk host contaminations and enrich microbial signals (Dalal et al. 2018). Although constrained by low throughput, limited DNA yield and the frequent need for subsequent amplification such as whole genome amplification, LMD offers a unique opportunity to connect microbial communities with host tissue microenvironments.

Selective lysis offers another route, exploiting differential vulnerabilities between host and microbial cells. While mammalian cells, lacking cell walls, are readily lysed with mild detergents (e.g., saponin, Triton X‐100, Tween 20) (Bruggeling et al. 2021; Hasan et al. 2016), plant cells present a major challenge due to their rigid cell walls composed of cellulose, hemicellulose and pectin (Rodrigues Mota et al. 2018). Saponin has shown promise in selectively lysing mammalian host cells without damaging bacterial cells (Aja‐Macaya et al. 2023; Charalampous et al. 2019; Ji et al. 2020). Following host cell lysis, DNase digestion is applied to degrade the released host DNA, leaving intact microbial cells for subsequent DNA extraction (Figure 2B). Several commercial kits, such as QIAamp DNA Microbiome Kit, Zymo HostZERO Microbial DNA Kit and MolYsis Complete5/Molzym Ultra‐Deep Microbiome Prep Kit, based on selective lysis and nuclease treatments, have been developed, effectively reducing host contamination in mammalian samples (Ong et al. 2022; Rajar et al. 2022; Yap et al. 2020). However, due to the structural complexity of plant cell walls, these kits cannot be directly applied to plant tissues. To address this, hydrolytic enzymes such as cellulase, hemicellulase and pectinase have been explored to degrade plant cell walls while leaving microbial cells intact (Figure 2C) (Jiao et al. 2006). However, the structure and composition of plant cell walls vary widely among species and tissues, necessitating customised optimisation of enzyme concentration and incubation conditions, limiting the development of universal protocols.

While chemical selective lysis combined with background DNA degradation can reduce host contamination to some extent in mammalian contexts, a notable drawback is the preferential lysis due to differing sensitivities of microbial species to lysis conditions, resulting in incomplete recovery and enrichment of certain microbial types. This method has limited efficiency on solid tissue samples (Wu‐Woods et al. 2023). Mechanical lysis methods, such as bead beating, offer an alternative by selectively disrupting larger host cells based on size differences, with bacterial cells being generally smaller (0.5–5 μm) than plant or mammalian host cells. By tuning bead size, shaking speed and duration, host cells can be preferentially lysed while preserving microbial integrity. For instance, using larger grinding beads (1.4 mm) to selectively disrupt host cells followed by appropriate DNases to degrade the released host DNA that effectively reduced host DNA contamination by over 1000‐fold, enabling the assembly of high‐quality bacterial MAGs (Wu‐Woods et al. 2023).

Following host cell lysis, enzymatic degradation of released DNA is critical. While traditional DNases are effective at degrading extracellular DNA (Bruggeling et al. 2021; Hasan et al. 2016), their activity can be influenced by factors such as pH, ionic strength and the presence of detergents, which can compromise their efficiency. Benzonase Nuclease, a broad‐spectrum endonuclease that cleaves both DNA and RNA, is favoured for host DNA degradation due to its wide operational range and high specificity (Nelson et al. 2019; Wu‐Woods et al. 2023). Additionally, the Heat Labile‐Salt Active Nuclease (HL‐SAN), a non‐specific endonuclease with optimal activity at high salt conditions, can also be employed to degrade released host DNA (Aja‐Macaya et al. 2023; Charalampous et al. 2019; Han and Xia 2023). An alternative approach to enzymatic degradation is the use of propidium monoazide (PMA), a cell membrane‐impermeable DNA intercalator. PMA covalently modifies extracellular DNA or DNA in dead cells, preventing its amplification (Nocker et al. 2006; Wang et al. 2021). This method has been shown to reduce host DNA contamination in human saliva samples without the need for nucleases (Marotz et al. 2018). However, PMA treatment showed negative impacts on DNA quality and library preparation in a bovine hindmilk microbiome study (Duarte and Porcellato 2024). It has also been reported that enzyme‐based lysis followed by PMA treatment can introduce dose‐dependent biases against Gram‐negative bacteria (Ganda et al. 2021).

Although differential lysis combined with DNase degradation is widely used and commercially available for host DNA removal, it often leaves insufficient microbial DNA for sequencing in low‐biomass samples. In these cases, whole genome amplification (WGA) through multiple displacement amplification (MDA) can be utilised to amplify raw DNA from nanograms to micrograms, ensuring adequate amounts for sequencing (Shi, Shao, et al. 2019). For instance, root endophytic bacteria have been enriched through density gradient centrifugation and single‐cell sorting, followed by MDA to generate single‐amplified genomes (Utturkar et al. 2016). A limitation of MDA is its propensity to introduce amplification biases, and any microbial or cross‐contamination in the sample can be exacerbated. An alternative method, primary template‐directed amplification (PTA), capitalises on the strong strand displacement activity and low error rate of phi29 polymerase. This technique employs exonuclease‐resistant terminators, producing smaller, double‐stranded amplification products that undergo limited subsequent amplification. This approach converts the reaction from exponential to a quasilinear process, facilitating more amplification from the primary template and reducing error propagation from daughter amplicons (Gonzalez‐Pena et al. 2021).

### 3.2 Removal of Host DNA After DNA Extraction

**FIGURE 3:**
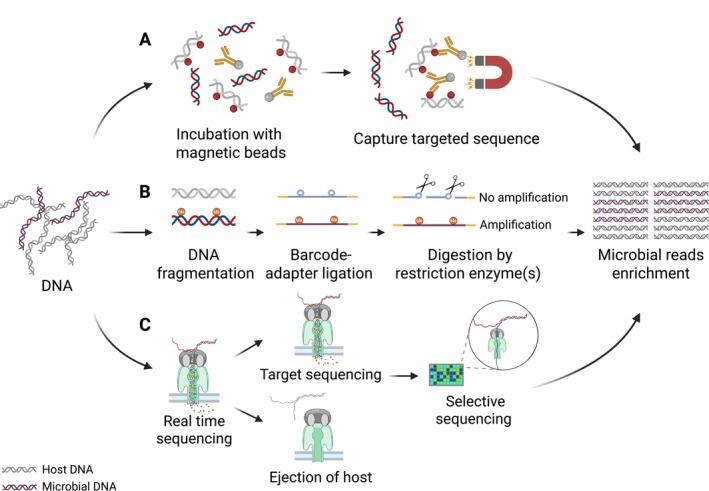
Host DNA depletion methods utilised post‐DNA extraction to enrich microbial reads in metagenomic samples. (A) Targeted sequence capture through incubation with magnetic beads, which bind specific microbial or host sequences to selectively enrich microbial DNA. (B) Restriction enzyme digestion targeting the differences in methylation motifs to impede the amplification of host DNA. (C) Nanopore selective sequencing technology, which ejects host DNA reads in real‐time, allowing for a higher concentration of microbial reads.

Significant sequence differences between host and microbial DNA can be leveraged for selective enrichment of microbial DNA after extraction. The development of highly multiplexed sequence capture techniques has enabled the targeted enrichment of DNA from hundreds of known viruses and bacteria by selectively amplifying their sequences (Xia et al. 2023). These probes can be immobilised on solid carriers or biotinylated for sequence‐specific hybridization with the target DNA (Figure 3A). However, a key limitation of microorganism‐specific hybridization capture is that it requires prior knowledge of the target DNA sequence to design the probes (Gasc et al. 2016). Alternatively, probes can be designed to target the host genome, allowing the study of entire microbial communities containing microorganisms with known or unknown sequences. Nevertheless, this hybridization‐based approach is often inefficient due to the complexity and large size of the host genome (Regalado et al. 2020).

DNA methylation, a key epigenetic modification, differs markedly between eukaryotes and prokaryotes in patterns, functions and biological effects. In eukaryotes, methylation primarily occurs on cytosines within CpG islands, forming 5‐methylcytosine (5mC) (de Mendoza et al. 2020). In prokaryotes, methylation occurs on adenine (N6‐methyladenine, 6mA) and cytosine (N4‐methylcytosine, 4mC), with 5mC being less common (Seong et al. 2021). Taking advantage of differences in CpG methylation density, engineered methyl‐CpG binding domains immobilised on paramagnetic beads can selectively capture and remove methylated host DNA (Feehery et al. 2013), as they specifically bind to double‐stranded DNA containing 5‐methyl CpG dinucleotides (Gebhard et al. 2006). This principle underlies the NEBNext Microbiome DNA Enrichment Kit, which has been widely used. However, some studies have reported low microbial enrichment efficiency using this method (Heravi et al. 2020; Marotz et al. 2018). Additionally, derivatives of human CXXC finger protein‐1, which have a specific affinity for non‐methylated CpG dinucleotides (Xu et al. 2011), have been employed to enrich prokaryotic DNA as LOOXSTER Enrichment Kit (Glassing et al. 2015).

Several alternative methods exploit bacterial DNA methylation motifs to differentiate microbial taxa from host background DNA. In prokaryotes, DNA methylation protects the bacterial genome from cleavage by restriction enzymes (Anton and Roberts 2021), a mechanism that can be utilised to selectively preserve bacterial DNA while degrading host DNA. Methylation‐sensitive restriction enzymes (e.g., XapI, DpnII) can be applied individually or in combination to digest non‐methylated host and background microbial DNA, thereby preserving the genomic DNA of bacterial taxa of interest (Figure 3B) (Cao et al. 2024; Enam et al. 2023). Nevertheless, the effectiveness of these methods is constrained by the limited characterisation of bacterial methylomes and the lack of methylation information for other microbial groups such as fungi, protozoa and viruses, potentially resulting in biased microbial recovery and skewed community profiles.

Nanopore sequencing, developed by Oxford Nanopore Technologies, enables real‐time, long‐read DNA sequencing by monitoring changes in electrical current as DNA molecules pass through a nanopore (Branton et al. 2008; Zhang et al. 2024). Unlike traditional short‐read sequencing platforms such as Illumina, nanopore technology facilitates rapid data acquisition and analysis, offering unique advantages for on‐site and time‐sensitive applications (Quick et al. 2016). A distinctive feature of nanopore sequencing is its selective sequencing capability, enabled by the ReadUntil technique, which allows real‐time classification of DNA molecules to determine whether to continue sequencing or eject unwanted molecules based on computational analysis of electrical signals (Loose et al. 2016) (Figure 3C). Tools such as UNCALLED (Kovaka et al. 2021) achieve fast mapping by directly aligning raw nanopore electrical signals to reference sequences without base calling, whereas Readfish (Munro et al. 2025; Payne et al. 2021) performs rapid base calling and subsequent sequence‐space mapping to guide selective sequencing decisions. Adaptive sampling strategies have been successfully applied to enrich rare species within metagenomic samples (Martin et al. 2022). Building upon these approaches, the recently developed method metaRUpore has demonstrated the effective enrichment of rare and unknown species from complex microbiomes using nanopore adaptive sampling (Sun et al. 2023). Moreover, selective sequencing has been successfully applied to host DNA depletion, resulting in a 1.7‐fold increase in total sequencing depth and improved taxonomic profiling sensitivity (Marquet et al. 2022). However, to date, no specialised tools have been developed specifically for direct microbial DNA enrichment in plant‐associated samples, highlighting an important area for future innovation.

## 4 Challenges and Opportunities

Plant microbiome research offers significant opportunities to enhance crop productivity, resilience and agricultural sustainability (Ge and Wang 2025). While recent advances in metagenomics have enabled a shift from descriptive taxonomic profiling to mechanistic insights into plant–microbe interactions (Gao et al. 2023), the pervasive presence of host‐derived DNA remains a critical bottleneck, particularly from low‐biomass tissues such as the phyllosphere and endosphere. Although several strategies are emerging, the efficiency and specificity of current host depletion methods remain major concerns, with many strategies struggling to balance effective host DNA removal and the preservation of microbial sequences. The difficulty is further compounded by the abundance of organellar DNA from chloroplasts and mitochondria, which often mimics bacterial genomic characteristics in size, GC content and sequence features, leading to the overrepresentation of host reads and masking of microbial signals (Lundberg et al. 2012, 2013). To mitigate this, bioinformatic tools such as reference‐based filtering and taxonomic classification approaches (e.g., Kraken2) are widely used to remove contaminating sequences post hoc, yet their effectiveness heavily depends on the availability of high‐quality reference genomes.

Additionally, various host depletion techniques may introduce biases that favour or exclude certain microbial taxa, potentially skewing the observed composition of microbial communities (Ahannach et al. 2021). These biases complicate comparative analyses and can lead to misinterpretations of metagenomic data. Moreover, the effectiveness of host depletion methods varies significantly across different sample types, including plant compartments and species, making the development of universally effective approaches challenging and necessitating sample‐specific optimization. Therefore, accurate quantification of microbial load is crucial for evaluating the efficiency of host DNA depletion strategies and interpreting metagenomic data. Several strategies are available for achieving microbial quantification. The use of internal spike‐in controls, such as synthetic DNA fragments or known microbial standards, enables absolute quantification of microbial content (Hardwick et al. 2018). Additionally, quantitative PCR (qPCR) and digital PCR (dPCR) targeting universal microbial genes (e.g., 16S rRNA genes) can be applied for quantifying the absolute abundance of microbial taxa (Barlow et al. 2020; Jian et al. 2020; Wu‐Woods et al. 2023). Integrating these quantitative approaches for developing host depletion approaches will improve the robustness, comparability and biological interpretation of plant metagenomic studies.

As metagenomic studies expand, there is an increasing demand for high‐throughput, cost‐effective host depletion techniques. Many current techniques are too labor‐intensive or expensive for large‐scale applications. Additionally, our limited understanding of the vast microbial diversity restricts the development of comprehensive and universally applicable depletion strategies. While most host removal methods have been optimised for mammalian samples, there is an urgent need to adapt and develop effective approaches specifically for plant samples. For instance, differential lysis approaches that leverage various bead sizes for mechanical disruption have shown promise in selectively lysing host cells and could be further optimised for diverse plant tissues and compartments (Wu‐Woods et al. 2023). Tailoring enzymatic treatments that selectively degrade plant cell walls and fine‐tuning conditions for specific plant compartments may further enhance microbial enrichment while minimising host DNA contamination.

In addition to experimental approaches, computational tools are invaluable for resolving host‐associated microbiome (Bai, Chen, et al. 2025; Bai, Ma, et al. 2025; Liu, Chen, et al. 2023; Peng et al. 2025). Several pipelines have been designed to computationally remove host DNA contamination, offering alternative or complementary strategies to laboratory‐based depletion methods (Constantinides et al. 2023; Hall and Coin 2024; Rumbavicius et al. 2023; Schmieder and Edwards 2011). These tools can significantly improve the accuracy and efficiency of metagenomic analyses, particularly when paired with high‐quality reference genomes and careful tool selection (Gao et al. 2025). Meanwhile, the emergence of artificial intelligence and machine learning may offer transformative opportunities to improve host DNA depletion strategies. Reference‐independent classifiers based on machine learning have shown success in distinguishing microbial from host reads with high accuracy (Krishnamoorthy et al. 2022). As these algorithms continue to evolve, they are expected to play an increasingly prominent role in host sequence discrimination (Liang et al. 2020). The integration of AI‐driven approaches into host depletion pipelines holds great promise for enhancing specificity, scalability and operational efficiency, ultimately unlocking new opportunities for comprehensive and high‐resolution plant microbiome analyses.

**FIGURE 4:**
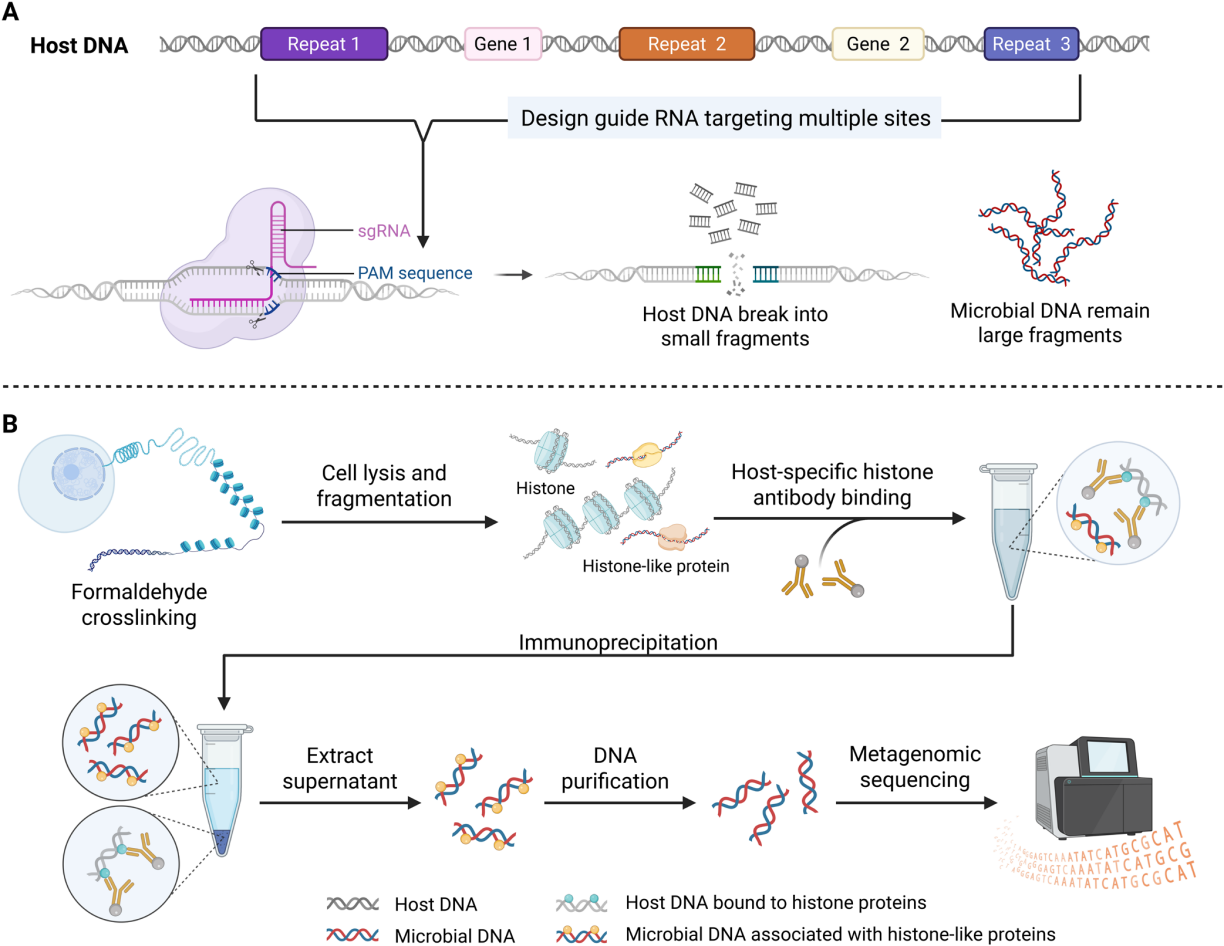
Potential novel methods for host DNA depletion in metagenomic samples. (A) CRISPR‐Cas9‐based host DNA removal: This approach leverages sequence differences between host and microbial DNA. Small‐guide RNAs (sgRNAs) are designed to target specific regions of the host genome, such as repetitive elements or highly expressed genes, for precise cleavage by the Cas9 enzyme. This results in the fragmentation of host DNA into small pieces, while leaving microbial DNA as long fragments, enabling its enrichment and further analysis. (B) Chromatin immunoprecipitation‐based host DNA depletion: This method exploits differences in chromatin structure between host and microbial cells. Formaldehyde crosslinking preserves the structure of chromatin by creating stable bonds between DNA and associated proteins. After cell lysis and DNA fragmentation, host‐specific histone antibodies are used to selectively bind and immunoprecipitate the host chromatin. The unbound fraction (primarily microbial DNA) is recovered from the supernatant and further purified for metagenomic sequencing.

Beyond current methods, we propose two potential strategies that offer promising new avenues for improving host DNA depletion while preserving microbial DNA integrity. One such approach leverages the CRISPR‐Cas system (Gu et al. 2016). Song et al. demonstrated that the Cas9 nuclease, combined with specific guide RNA (gRNA), can target and cut rice 16S rRNA gene without significant off‐target effects, reducing host contamination in amplicon sequencing (Song and Xie 2020). CRISPR‐based depletion approaches have also been used to reduce host RNA and enhance microbial enrichment in pandemic diagnostics (Chan et al. 2023). By designing gRNAs to target repetitive or high‐copy regions of the host genome, Cas9 can fragment host DNA into short pieces (e.g., 1–2 kb), leaving microbial DNA as long fragments for long‐read sequencing (Figure 4A). The feasibility of this approach is supported by the availability of high‐quality plant reference genomes, facilitating accurate and high‐throughput gRNA design using tools such as CRISPR‐PLANT v2 (Minkenberg et al. 2019) and CRISPR‐P 2.0 (Liu et al. 2017). Nevertheless, the strategy faces technical challenges. Comprehensive host depletion across large and complex plant genomes may require a large pool of gRNAs to achieve adequate coverage. This introduces substantial gRNA design complexity, as achieving efficient cleavage while minimising off‐target effects requires balancing gRNA number, distribution, sequence specificity and compatibility with Cas9 activity (Huang et al. 2022; Liu, Zhang, et al. 2023). Moreover, gRNA targets must be carefully selected to avoid unintended cleavage of microbial DNA. When implemented effectively, this method is especially advantageous for long‐read metagenomic sequencing platforms such as Oxford Nanopore and PacBio, which preferentially sequence longer DNA fragments and are less efficient at reading short fragments. The fragmented host DNA may be excluded by size selection, library preparation or data processing. This size bias can be exploited to enrich microbial sequences following CRISPR‐based host DNA fragmentation, thereby improving the efficiency and accuracy of downstream metagenomic analyses.

Another promising approach is chromatin immunoprecipitation (ChIP)‐based host DNA depletion, which exploits structural differences between host and microbial chromatin. Formaldehyde crosslinking preserves chromatin structure by creating stable bonds between DNA and associated proteins, thereby stabilising host chromatin. After cell lysis and controlled fragmentation, anti‐histone antibodies can more effectively and selectively bind to host chromatin, enabling its immunoprecipitation. Microbial DNA, which is generally not associated with histones, remains in the unbound supernatant and can be purified for metagenomic sequencing (Figure 4B). The success of this technique depends largely on the availability of broad‐spectrum antibodies that can selectively bind host histones without cross‐reacting with microbial histone‐like proteins. Further optimisation of crosslinking conditions, fragmentation strategies and antibody specificity will be critical for adapting this technique to a wide range of plant tissues and microbial communities.

## 5 Conclusion

Understanding plant–microbe interactions is fundamental for advancing sustainable agriculture, improving crop resilience and promoting ecosystem health (Russ et al. 2023). While metagenomics has revolutionised the study of plant microbiomes by enabling comprehensive and culture‐independent analyses of microbial community structure and function (Dai et al. 2025), its application to low‐biomass plant compartments, such as the phyllosphere and endosphere, remains limited. This is primarily due to high levels of host DNA contamination, which can obscure microbial signals and compromise sequencing efficiency. Only through efficient and specific depletion strategies can we fully harness metagenomics to reveal the complete breadth of plant‐associated microbial ecosystems and their functional contributions to agricultural resilience.

In this review, we summarised the current strategies for addressing host DNA contamination, including physical separation, selective lysis and sequence‐based enrichment approaches, each with its own advantages and limitations. Due to the diverse nature of plant tissues and associated microbial communities, these approaches often require tailored optimisation based on sample type and research objectives. Additionally, we propose two novel conceptual approaches for host DNA depletion: CRISPR‐Cas9‐mediated host genome cleavage and chromatin immunoprecipitation‐based host DNA removal. Although requiring experimental validation, these methods offer promising directions for enhancing microbial DNA enrichment and minimising host interference in host‐derived samples. Besides, computational tools complement experimental methods by further reducing host contamination during downstream analyses and improving data interpretation. The rapid advancement of AI‐driven approaches is expected to accelerate the development of more efficient and precise host depletion strategies (Bai, Chen, et al. 2025; Bai, Ma, et al. 2025; Liu, Chen, et al. 2023; Peng et al. 2025).

**FIGURE 5:**
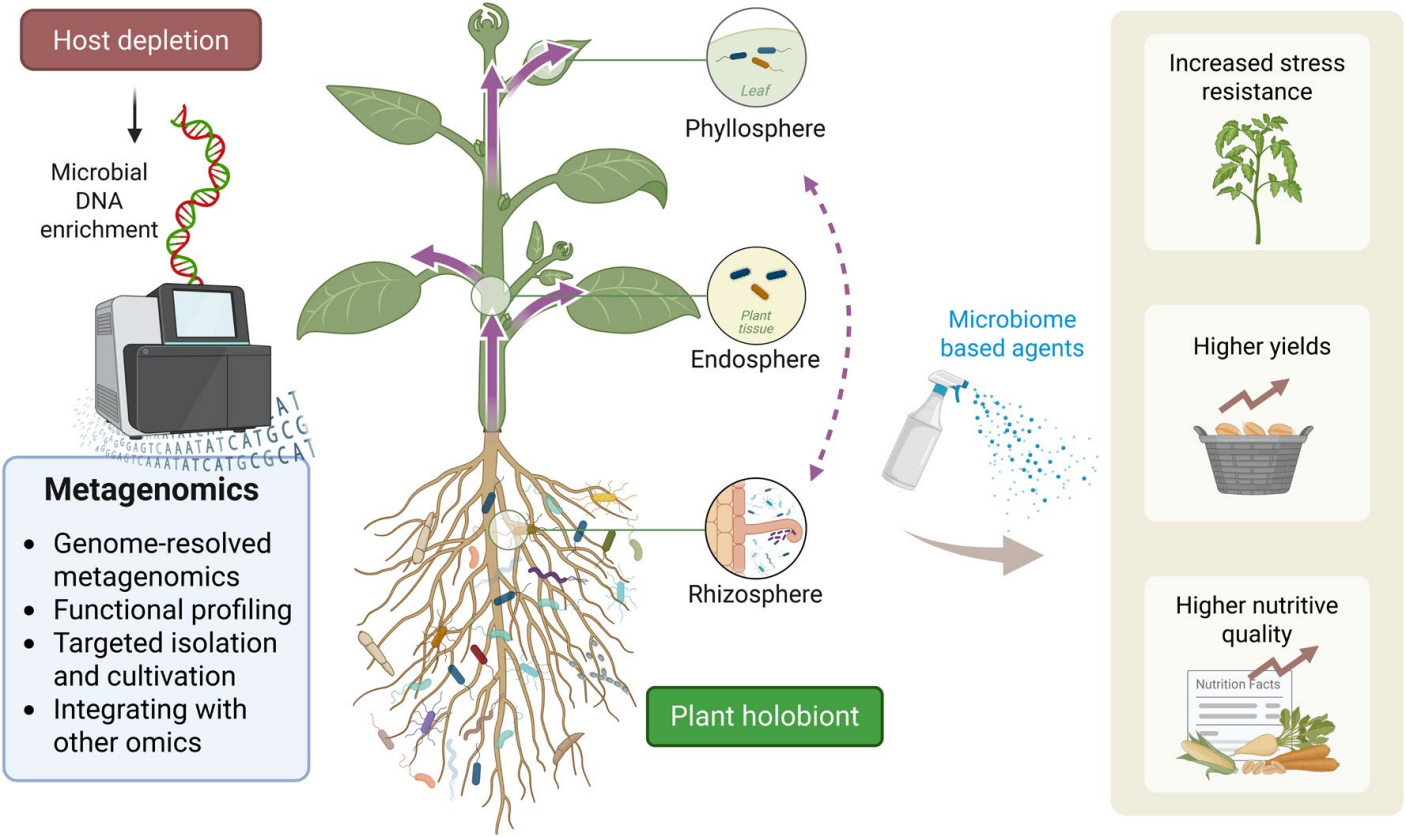
Host DNA depletion facilitates metagenomic analysis of the plant holobiont and enables microbiome‐based applications in sustainable agriculture. Effective host depletion methods enrich microbial DNA from plant‐associated samples, improving the resolution and accuracy of metagenomic analyses. This supports genome‐resolved metagenomics, functional profiling and integration with other omics (e.g., metatranscriptomics, metabolomics), thereby enabling a holistic understanding of plant microbiomes across compartments such as the rhizosphere, endosphere and phyllosphere. Insights gained can inform the development of targeted microbiome‐based agents for crop management, ultimately contributing to increased stress tolerance, improved yields and enhanced nutritional quality of crops.

Refining and standardising host depletion methods will be pivotal for extending the reach of metagenomics into previously underexplored plant niches. More efficient and specific depletion techniques will enable researchers to access high‐quality microbial genomic data, deepen functional analyses and uncover the ecological dynamics of plant‐associated microbial communities (Nayfach and Pollard 2016). Importantly, metagenomics is driving advances across multiple domains of plant microbiome research. Genome‐resolved metagenomics enables reconstruction of high‐quality microbial genomes directly from environmental samples, providing insights into uncultured taxa (Almeida et al. 2019; Dai et al. 2025; Parks et al. 2017). Functional profiling reveals the metabolic potential of microbial communities, guiding the development of tailored microbial consortia (García‐Jiménez et al. 2018; Jing et al. 2024; Mataigne et al. 2022). Metagenomic data can also inform targeted isolation and cultivation (e.g., reverse genomics approaches) to access novel taxa (Armetta et al. 2025; Cross et al. 2019). Furthermore, integrating metagenomics with other omics approaches—including metatranscriptomics, metaproteomics and metabolomics—offers a systems‐level understanding of microbial activity and function (Jansson and Baker 2016; Pedersen et al. 2018). This multi‐omics integration allows researchers to connect genomic potential with transcriptional regulation, protein expression and metabolite exchange, thereby elucidating how microbial communities dynamically respond to environmental and host‐related cues (Figure 5) (Chen et al. 2025; Getzke et al. 2023; Ofek‐Lalzar et al. 2014; Vannier et al. 2023; Wolinska et al. 2021).

These integrative strategies have deepened our understanding of plant–microbe interactions and contributed to the development of biological control agents, biofertilizers and microbiome‐based crop management strategies that reduce reliance on chemical inputs and enhance ecosystem resilience (Li et al. 2024; Trivedi et al. 2021). For instance, beneficial microbes such as strains of *Pseudomonas*, *Bacillus* and arbuscular mycorrhizal fungi have been commercially deployed as biocontrol agents to promote plant growth and suppress pathogens. Synthetic microbial communities are rapidly emerging as powerful tools in agriculture, offering promising applications by leveraging cooperative microbial traits and functional complementarity (Karmakar et al. 2025; Martins et al. 2023; Northen et al. 2024; Vorholt et al. 2017). More recently, microbiome‐based breeding strategies have been proposed, aiming to rationally manipulate root traits to selectively recruit beneficial microbes or deter pathogens, thereby optimising plant performance through targeted host–microbiome interactions (Cernava 2024; Ge and Wang 2025; Nerva et al. 2022). Collectively, these advancements underscore the transformative potential of plant microbiome research in shaping next‐generation, microbe‐based agricultural solutions that promote crop resilience and productivity under increasingly variable environmental conditions (Afridi et al. 2022; Copeland et al. 2025; French et al. 2021).

Ultimately, overcoming host contamination barriers enables a shift toward a holistic, holobiont‐level view—considering the plant and its microbiota as an integrated ecological unit (Vandenkoornhuyse et al. 2015; Zilber‐Rosenberg and Rosenberg 2008). This holistic view enables the investigation of spatially distributed microbial communities across different plant compartments and their coordinated roles in plant development, metabolism and immunity. Embracing such a systems‐level approach is essential for deciphering complex plant–microbiome interactions and for driving innovation in sustainable agriculture, ecosystem resilience and global food security.

## Author Contributions

Yao Wang conceived the review framework and wrote the manuscript. Junbo Yang, Huiyu Hou, Luyang Song and Xu Cheng revised the manuscript. Yong‐Xin Liu provided critical guidance, supervision and funding support. All authors have read the final manuscript and approved it for publication.

## Conflicts of Interest

The authors declare no conflicts of interest.

## Data Availability

This study did not generate any new data. All data supporting the findings of this review are available in the cited literature.
